# Embodied neurofeedback with an anthropomorphic robotic hand

**DOI:** 10.1038/srep37696

**Published:** 2016-11-21

**Authors:** Niclas Braun, Reiner Emkes, Jeremy D. Thorne, Stefan Debener

**Affiliations:** 1 Neuropsychology Lab, Department of Psychology, University of Oldenburg, Oldenburg, Germany; 2 Research Center Neurosensory Science, University of Oldenburg, Oldenburg, Germany; 3 Cluster of Excellence Hearing4All, University of Oldenburg, Germany

## Abstract

Neurofeedback-guided motor imagery training (NF-MIT) has been suggested as a promising therapy for stroke-induced motor impairment. Whereas much NF-MIT research has aimed at signal processing optimization, the type of sensory feedback given to the participant has received less attention. Often the feedback signal is highly abstract and not inherently coupled to the mental act performed. In this study, we asked whether an embodied feedback signal is more efficient for neurofeedback operation than a non-embodiable feedback signal. Inspired by the rubber hand illusion, demonstrating that an artificial hand can be incorporated into one’s own body scheme, we used an anthropomorphic robotic hand to visually guide the participants’ motor imagery act and to deliver neurofeedback. Using two experimental manipulations, we investigated how a participant’s neurofeedback performance and subjective experience were influenced by the embodiability of the robotic hand, and by the neurofeedback signal’s validity. As pertains to embodiment, we found a promoting effect of robotic-hand embodiment in subjective, behavioral, electrophysiological and electrodermal measures. Regarding neurofeedback signal validity, we found some differences between real and sham neurofeedback in terms of subjective and electrodermal measures, but not in terms of behavioral and electrophysiological measures. This study motivates the further development of embodied feedback signals for NF-MIT.

Stroke is the leading cause of chronic motor impairment in adults, often resulting in hand weakness and loss of upper limb fine motor skills^1^. To aid upper limb motor recovery, various interventions have been developed^2^. Constrained induced movement therapy is among the most thoroughly evaluated and most effective interventions. It encourages goal-directed, highly-repetitive movement with the paretic limb, while constraining the non-affected limb^3^,^4^. It is, however, only applicable in patients with residual motor skills. For fully plegic patients, alternative interventions are needed, such as mirror visual feedback (MVF)^5^ or neurofeedback-guided motor imagery training (NF-MIT)^6^. These interventions do not require residual motor function, as they address motor recovery through mere motor simulation. Based on neurofunctional evidence demonstrating similar cortical activation patterns during motor execution and motor simulation, the common goal of these approaches is to induce motor-specific brain plasticity and reverse maladaptive, non-use based reorganization patterns, thereby contributing to motor recovery^5^,^7^,^8^.

In MVF, the motor percept is bottom-up induced by the sensory stimulation of visual motion feedback^5^. The patient’s healthy limb is placed in front of a mirror with the paretic counterpart behind it, such that from the patient’s view, the healthy limb’s mirror reflection is superimposed on the paretic limb^5^,^9^,^10^. When asked to conduct predefined movements with the healthy limb and attend to the mirror reflection, many patients report a vivid movement illusion, in that they experience the paretic limb to be moving again. Apparently, the illusory experience of a sense of ownership (SoO) and sense of agency (SoA) for the moving hand in the mirror may transiently replace the paretic limb experience. The illusion however diminishes as soon as the healthy limb stops moving, or is pulled away. In classical MVF, limb motion feedback thus can only occur in the presence of concomitant movements with the contralateral, non-affected limb^5^. It is therefore also desirable to develop training regimes that provide unilateral isolated limb motion feedback without a mirror. Inspired by the rubber hand illusion (RHI)^11^, which shows that visuo-tactile stimulation can induce a SoO over a static artificial hand, Caspar *et al.*^12^ developed an active RHI variant, in which limb motion feedback is provided via the movements of an anthropomorphic robotic hand. Likewise, several virtual reality studies have been reported, in which limb motion feedback is provided via head-mounted displays^13^,^14^.

In NF-MIT, the desired motor percept is top-down generated by cognition, since here the participants self-induce the motor percept by mental rehearsal alone^15^. The online feedback is typically based on event-related desynchronization (ERD), a MI-related decrease in 8–30 Hz oscillatory brain activity over the sensorimotor areas^16^. While popular in the context of brain-computer interfaces, the type of sensory feedback provided to the participant during MI has received less attention. In most NF-MITs, the feedback signal is rather abstract and not inherently coupled to the mental act performed^17^. Only recently, virtually-presented limbs, ortheses or rehabilitation robots have been used to convey the neurofeedback signal^18^,^19^,^20^,^21^,^22^, but how strongly these setups induce SoO and SoA, and how important these concepts are for NF-MIT is poorly understood.

As stated above, in NF-MIT the motor percept is self-constructed by mental imagery whereas in MVF it is bottom-up induced by the sensory input. Whereas in MVF, much of the cognitive workload can be offloaded onto the mirror, more mental effort is required in NF-MIT^23^. Given the fact, that stroke-induced motor impairment seldom comes in isolation, but is typically accompanied by cognitive impairment^24^, sensibility loss^25^,^26^ or MI ability impairment^27^, the question thus is which patients are actually still able to perform MI with their paretic body side^28^,^29^, and when is a bottom-up motor percept induct more applicable. Whereas in NF-MIT the patient is supported by a neurofeedback signal, which helps to perform the mental act and facilitates imagery-induced motor cortex activation^30^,^31^, no neurofeedback signal can typically be provided in MVF. The aim of the present study was therefore to develop a new NF-MIT that integrates the positive aspects of MVF. Inspired by the RHI, we developed an anthropomorphic robotic hand to visually guide the participant’s MI act and deliver embodied neurofeedback. We predicted that an embodied feedback signal closely resembling the mental act performed should be more intuitive for neurofeedback operation than non-embodiable feedback and therefore should also lead to a better NF-MIT performance^18^. As an artificial hand is known only to induce SoO, if it is placed in an anatomically-congruent position^32^,^33^,^34^, we tested the hypothesized benefit of embodied feedback by comparing neurofeedback delivered by the artificial hand in a congruent position with that delivered in an incongruent position. We also asked whether NF-MIT performance and SoA depend on the validity of the neurofeedback signal. This was done by including a sham feedback condition in which the provided feedback was not based on real-time brain activity.

## Materials and Methods

### Participants

Twenty-five participants (9 females; all aged 20–30) were recruited for the study. Individuals were required to have normal or corrected-to-normal vision, a relatively large hand size (hand breath >10 cm; hand length >17.5 cm), no known history of psychiatric or neurological disorders, and were not taking psychoactive medication. All participants gave written informed consent, were paid for their participation and were naive to the purpose of the study. In addition, none of the participants had previous experience with neurofeedback or the RHI. The experiment was conducted in accordance with the Declaration of Helsinki and was approved by the University of Oldenburg ethics committee. Four participants had to be excluded from the statistical analysis, three due to technical malfunctioning and one for failing to follow task instructions.

### Overview

The full experiment consisted of two recording sessions conducted on two separate days within one week. On the first day, several questionnaires were administered and participants became acquainted with the overall MI task (see section training phase). On the second day, the actual NF-MIT took place.

### Apparatus

**Figure 1:**
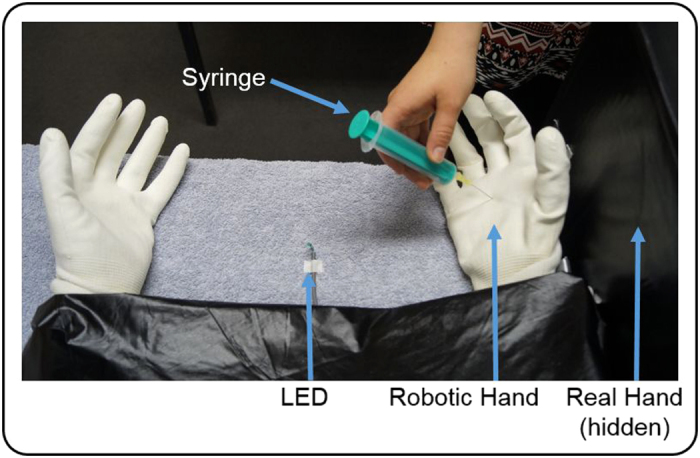
Study design. As in the classical rubber hand illusion, participants placed their right hand into a black box, whereas the robotic hand was placed in direct vision in front of the participant. During training, participants kinesthetically imagined flexion-extension movements in spatio-temporal synchrony to the flexion-extension movements of the robotic hand. A small LED thereby announced the beginning of each new MI trial. During feedback, the participants again imagined flexion-extension movements. This time, however, the robotic hand only moved, if the classification algorithm detected the participant’s momentary MI brain state, or 5 seconds were over. The neurofeedback loop was thus implicitly encoded in the robotic hands movements. To test whether the participants had incorporated the robotic hand into their own body schema, a syringe was pricked into the robotic hand after each feedback run, and the participant’s subjective level of authorship and ownership towards the robotic hand was documented.

The experimental set-up was adapted from the RHI-paradigm^11^ and is depicted in Fig. 1. The participant sat in front of a rectangular table (50 × 60 cm), resting both arms on a table. The right hand and lower arm were covered with a black box so that they were not visible. The anthropomorphic robotic hand was placed directly medially aside the participant’s real right hand, adjacent to the black box. Depending on the conditions, the robotic hand was placed in either an anatomically correct position (congruent condition) or was rotated by 180°, with the fingers pointing towards the participant (incongruent condition). The horizontal distance between the robotic hand and the participant’s real right hand was kept as small as possible and amounted to approximately 7.5 cm. The robotic hand was covered with a thin-gauge garden glove and the participants wore an identical glove on their right (unseen) hand. On the left side, the participants either also wore a glove, or the hand was covered by a towel. Throughout the experiment, a blanket covered the participant’s shoulders and arm and the space between the robotic hand and the participant’s body, thereby facilitating the visual impression that the robotic hand could be the participant’s own hand (see Fig. 1).

The robotic hand was assembled by one of the authors (NB) using mechanical components, mostly created using 3D printing technology based on an online 3D hand template^35^ (for further details, see http://inmoov.fr/hand-and-forarm/). Fully opened the robotic hand measured 18 cm in length. Effort was made in optimizing the robotic hand, aiming for both a realistic appearance and realistic motion behavior. The finally used version of the robotic hand closely resembled a typical male human hand in terms of shape and size, and natural open-close movements could be realistically mimicked. The robotic hand movements were mechanically realized by five small 6 V servos, one for each digit. The servos were located approximately 1 m away in a separate, sound shielded box. To prevent residual mechanical noise to be audible, participants wore earplugs throughout the experiment. The microcontroller (Arduino mega 2560) controlling the servos was connected to a control computer via serial port (baudrate: 9600), and allowed us to control the robotic hand movements directly from Matlab R2012a (Mathworks, Natick, MA, USA). A time delay of less than 200 ms was achieved between a Matlab motor command and actual robotic hand movement onset.

### Design

A 3 × 2 factorial design was used for the behavioral analysis and most remaining analyses were fractional subsets of this overall design. The two within-subjects factors were condition and phase. The condition factor consisted of the levels congruent, incongruent and sham, the phase factor of the levels training and feedback. The congruent level only differed from the incongruent level in the positioning of the robotic hand; it was either placed in anatomical congruency to the participant’s real hand, or rotated by 180°. The sham feedback condition was identical to the congruent feedback condition, except that here the provided neurofeedback was based on a replay of previously recorded data from the same participant (details below).

### Procedure

Day 1, as stated above, consisted of questionnaires and familiarization. For task familiarization, participants completed one full training phase (see below) for each condition. No neurofeedback, however, was provided. The experiment on day 2 took place in a dimly-lit, sound-attenuated recording booth. The experiment consisted of three experimental blocks, each of them following the same within-block structure. Each block began with a training phase where the participants conducted the MI task in temporal synchrony with computer-controlled robotic hand movements. This was done to allow the participants to perceptually bind together the mental act and the robotic hand movement. Afterwards a short structured interview was conducted, to document the participant’s subjective experience during this phase. After the interview, which lasted approximately three minutes, the feedback phase started. Here the participant continued with the same MI task as before, except that now the robotic hand movements depended on the current (or replayed) brain activity; or, more precisely, on the mental state estimations of the classification algorithm. Finally, immediately after the feedback phase, the experimenter came again into the recording booth and pricked the robotic hand with a medium-sized syringe (length of needle: 2.5 cm). Afterwards the same interview as following the previous block was conducted. Block order effects were counterbalanced across participants, although the sham block was never conducted before the congruent block. This was necessary, because in the sham feedback phase, the robotic hand’s movement behavior was implemented as a replay of the preceding congruent feedback phase.

### Training phase

The training phase was conducted on days 1 and 2. On day 1, it was used to acquaint the participant with the overall MI task. On day 2, a training phase (see section overview) was included not only for training the classifier, but also for RHI-induction and as a mental preparation for the subsequent feedback phase. To enable successful RHI induction and at the same time keep the trial structure between the training and feedback phase as similar as possible, robotic hand movements were included in the training phase. Apart from the experimental manipulation of the condition factor, each training phase was identically structured with a duration of 12 min and consisting of approximately 85 trials. Each trial lasted around 8.5 seconds and began with a jittered 4–6 seconds rest period during which the participant was instructed to relax. These values were chosen based on our lab experience, pilot data and former studies^30^,^31^. The robotic hand was in the open state during this period and did not move. After the rest period, a small LED indicated to the participant to prepare for the following MI period, which began 300 ms later. The MI period lasted 3 seconds. During the MI period, the robotic hand first flexed and immediately extended again its fingers, and the participant was required to concomitantly imagine the same movement with his or her right hand. One flexion-extension cycle of the robotic hand lasted 3 seconds. The instruction was to imagine the movement kinesthetically from a first person perspective, in spatio-temporal synchrony with the robotic hand movement.

### Feedback phase

The trials within the feedback phase were identically structured to the trials in the training phase, except that now the robotic hand movements depended on the classifier’s ongoing brain state estimations. During the rest period, no robotic hand movement occurred and the period was prolonged until the classifier detected the desired resting brain state, or 5 seconds had passed. Thus after a maximum of 5 seconds rest period prolongation, the LED was switched on again, cueing the beginning of the next MI period. During the MI period the robotic hand movement onset was delayed until the classifier detected the desired MI brain state, or alternatively, 5 seconds had passed. Afterwards the robotic hand started moving again, even if the classifier had not (yet) detected the MI brain state. This was done to keep the participants motivated even if the MI brain states could not be detected. Overall, the neurofeedback loop was thus implicitly encoded in the delay between the participant’s MI-act and the resulting robotic hand movement. The better the performance was, the shorter was the introduced time interval between the MI act and the robotic hand movement, and thus, the more favorable was the temporal congruency, which helps in inducing the RHI^33^,^36^.

### EEG-based feature extraction and classifier training

To enable neurofeedback, one classifier discriminating rest from MI periods was used for each feedback phase. Each classifier was based on the EEG data of the corresponding training phase and was calculated just before the start of the feedback phase. The classifier for the sham feedback phase was not used for online neurofeedback, but later used for performance evaluation. EEG data were collected from 59 scalp sites using an elastic cap (EASYCAP, Herrsching, Germany). Using two BrainAmp DC amplifiers (BrainProducts GmbH, Herrsching, Germany), the continuous EEG signal was digitized via Lab Streaming Layer (LSL; https://code.google.com/p/labstreaminglayer) with a sampling rate of 200 Hz. The nose-tip served as reference and a central fronto-polar electrode as ground. EEG data were analyzed with EEGLAB^37^ and BCILAB^38^. An adaptation of BCILAB’s pre-built ParadigmCSP class^38^ was used as the learning algorithm. In this paradigm, class-specific changes in the sensorimotor rhythm are extracted by means of common spatial pattern (CSP) analysis. Given two time windows of a multivariate signal, the CSP algorithm seeks to find spatial filters that maximize the variance for one class and simultaneously minimize the variance for the other class^39^,^40^. To derive these CSP filters, the EEG data were first split into one dataset containing rest period segments (last 2 seconds) and one dataset containing MI period segments (first 2 seconds). Artifactual trials (around 20%) were identified and rejected using built-in EEGLAB functions, because the CSP algorithm is known to be sensitive to outliers^39^. After artifact rejection, the two datasets were 8–30 Hz bandpass-filtered, and their covariance matrices were calculated. Defining 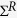 and 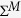 as the covariance matrices of the rest and MI segments, the CSP filters were derived by solving the following generalized eigenvalue problem:


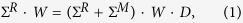


where 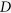 is a diagonal matrix containing the generalized eigenvalues of 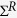 and 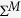 on its diagonal, and 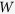 is a matrix containing the generalized eigenvectors of 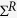 and 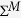, i.e. the CSP filters. 59 CSP filters were derived by this procedure and their corresponding CSP patterns by the pseudoinverse of *W*. The first four and last four CSP components (promising high class-discriminability) were manually inspected based on their spatial filter and pattern topography and associated time course. Physiologically plausible CSP components showing a clear ERD-pattern^41^ were kept (group average: 1.76 components) and CSP-filtered time series segments (CSP segments) were calculated by multiplying each EEG segment from the two datasets with each selected CSP-filter. The log-variance of each CSP segment was used as a feature value. The number of feature values in the feature vector was thus the same size as the number of CSP filters used. To obtain probabilistic class estimates, a regularized logistic regression model was used for classifying rest periods vs. MI periods.

### Online data flow and classification

For online data flow and classification during the congruent and incongruent feedback phase, LSL and BCILAB were used. EEG online data were acquired by LSL and accessed from within BCILAB. During the neurofeedback interval, the classifier was repeatedly consulted every 50 ms (operating on the most recent 2-second EEG segment) until it reliably detected the desired brain state, or 5 seconds had passed. Feature values were calculated as before during classifier training. To reduce the likelihood that the classifier randomly guessed the right state, the classifier vote for a correct brain state estimate was only accepted if its probability estimate was higher than 70%. For feedback provision during the sham feedback phase, the participant’s individual neurofeedback performance during the preceding congruent feedback phase was replayed. This resulted in near identical feedback between the congruent and the sham feedback condition.

### Performance evaluation

Online classification accuracies were calculated for all three feedback conditions using the same time windows as used for classifier training. In addition, sensitivity and specificity values were calculated. Sensitivity was defined as the percentage of correctly identified MI phases and specificity as the percentage of correctly identified rest phases. Moreover, for the congruent and incongruent feedback phases, the average detection time, which was defined as the time that elapsed from period onset until the classifier detected the desired brain state in the EEG, was separately documented for rest and MI periods. Only those trials were considered where the classifier detected the respective rest or MI period within 3 s (approx. 70% of the trials).

### Questionnaire data

A 10-item questionnaire was adopted from previous studies^32^,^33^ and used for the assessment of subjective experience (Table 1). The questions were read by the experimenter at the end of each block and the participants indicated their level of agreement on a 7-point Likert scale, ranging from −3 (“totally disagree”) to +3 (“totally agree”). Four phenomenal target properties were operationalized: The SoO, SoA, Experiential Realness (ER) and MI-action binding (MIAB). The SoO was defined as the illusory feeling of “mineness” towards the robotic hand and the SoA as the subjective amount of authorship over the robotic hand behavior. ER was defined as how vivid and real the own MI act was experienced and MIAB as how strongly the self-induced MI percept and the robotic hand motion percept felt being bound together. Two statements were used for each phenomenal target property (see Table 1) and later averaged to obtain a single value for each property, subject and condition. The remaining two statements served as control statements. One related to the SoA (“I felt as if the robotic hand were controlling my will”.) and one to the SoO (“I felt as if I no longer had a right hand, as if I my right hand disappeared”.). The control statements included illusion-related statements, but did not specifically capture the phenomenal experience of self-agency or limb ownership. Hence, with successful SoA induction, the SoA-related questions should have high affirmative ratings whereas the SoA control question should not be specifically affected by the respective experimental manipulation. Likewise, with successful SoO induction, the SoO related questions should have high affirmative ratings, whereas the ratings for the SoO-control question should remain unaffected. All statements appeared in a pseudo-randomized order. As in former studies^32^,^33^,^42^ the illusion criterion for the SoO and SoA was set to >= +1. Hence, an average score >= +1 was interpreted as affirmation of the respective SoO or SoA experience for the respective participant.

### EEG offline analysis

EEG offline analysis focused on temporo-spectral differences between the congruent and incongruent conditions. EEG data artifact attenuation was performed using extended infomax independent component analysis^37^,^43^ (ICA). The ICA-corrected data were segmented from −2.4 to 4.4 s, relative to the onset of the MI period. Segments containing unique, non-stereotyped artifacts were identified by built-in EEGLAB functions and rejected. From each remaining EEG segments, a corresponding CSP segment was calculated by multiplying the data with a chosen, cross-conditional CSP-filter. Cross-conditional CSP-components were used instead of condition-specific CSP components to avoid condition differences in the ERD that were due to different spatial filters. To obtain a unique CSP-component for each participant, all 59 potential CSP components were first derived from the congruent-train and incongruent-train phase segments (as described above) and then only the physiologically most plausible CSP component was kept. A time-frequency (TF) analysis was performed on the CSP segments. Using a continuous Morlet wavelet transform^44^,^45^, two-dimensional TF windows were calculated for each CSP segment. The obtained frequency bins ranged from 5 to 50 Hz in 1 Hz frequency steps. To avoid edge-artifacts, the original TF data were reduced from −2 s to +4 s relative to MI onset. Percent power change from baseline values were calculated for each pixel by squaring the vector length, scaling it to decibels (10 × log10) and calculating its change in power, relative to the mean power of its respective frequency bin between −2 s to −0.5 s. For the statistical analysis, ERD values were extracted for each condition and phase by taking the mean percent log power changes across trials between 10 and 25 Hz (the frequency range, where most of our participants showed the strongest desynchronization), averaged over a 2 seconds time interval beginning 250 ms after MI onset.

### Phasic electrodermal activity

Electrodermal activity (EDA) was recorded during syringe application and used as an implicit measure of robotic hand embodiment^46^,^47^,^48^. Based on previous work, we expected a stronger skin conductance response in the congruent than incongruent feedback phase. EDA recordings were conducted by following previously established criteria^49^. Two sintered silver/silver chloride (Ag/AgCl) electrodes were filled with a sodium chloride (NaCl) paste and were attached to the index and middle fingers of the right hand. Electrode contact areas were approximately 7 mm in diameter. The continuous EDA signal was analog filtered from 0 to 200 Hz and digitized with a sampling frequency of 200 Hz (0.006 μS resolution). EDA data were analyzed with the software LEDALAB v3.4.3^50^,^51^, which implements a continuous decomposition analysis (CDA). CDA deconvolves the data and decomposes phasic from tonic portions of EDA^50^,^51^. The resulting phasic EDA was considered and segmented from −6 s to +11 s relative to the application of the syringe. The segments were baseline-corrected by calculating the percent amplitude change, relative to the average EDA of the first 2-second time interval. For statistical analysis, the mean phasic EDA response (in %) was calculated for the +3 to +9 seconds interval, relative to syringe application.

### EMG analysis

To confirm that participants followed task instructions and avoided condition-specific limb movements EMG activity was recorded with two EMG channels (Musculus flexor digitorum superficialis, antecubital fossa) using a bipolar montage and compared between rest and MI phases. For each subject, single-trial RMS values were separately calculated for each rest and movement period and then a t-test was performed to explore statistical differences (α = 0.01) between conditions. Condition-specific movements were found in one subject (t(86) = −3.40; p < 0.001) who was consequently removed from further analysis.

### Statistical analysis

The experiment included eight dependent variables (accuracy, detection time, SoA, SoO, ER, MIAB, ERD and phasic EDA) and two control variables (SoO control scores, SoA control scores). To statistically test whether classification accuracies were above chance-level a binomial statistic with a confidence limit of p = 0.05 was used^52^. For the group analysis, classification accuracies were statistically analyzed by a nonparametric one-way Friedman test with the factor condition (congruent, incongruent, sham), and then followed up by pairwise Friedman comparisons. Detection times were analyzed by a two-way repeated measures ANOVA with factors condition (congruent vs. incongruent) and period (rest vs. MI). Phasic EDA responses were analyzed using a one-way repeated-measures ANOVA with the factor condition (congruent, incongruent, sham). For SoA and SoO and evaluation, control statements were available. These statements allowed us to test how specific the experimental manipulations were, that is, whether the manipulations only affected the illusion-specific statements or also those statements that went beyond the mere phenomenal experience of ownership or agency. To statistically validate this, t-tests were performed comparing the SoO values and SoO control values of the congruent, incongruent and sham training phases and the SoA and SoA control values of the congruent, incongruent and sham feedback phases. For each phenomenal target property (SoO, SoA, ER, MIAB), a two-way repeated measures ANOVAs with the two factors condition (congruent, incongruent, sham) and phase (training vs. feedback) was conducted. Where applicable, interaction effects were followed up by pairwise t-tests. Additionally, to study the relationship between these measures, correlation coefficients were calculated across condition levels for each possible pair (21) of measures. The coefficients were obtained by calculating the mean value across conditions for each variable and participant, and then calculating the correlation coefficient for these mean values. To avoid type 1 errors due to multiple comparisons, Bonferroni correction was applied (alpha = 0.05/21 = 0.0024).

## Results

### Classification accuracies

**Figure 2:**
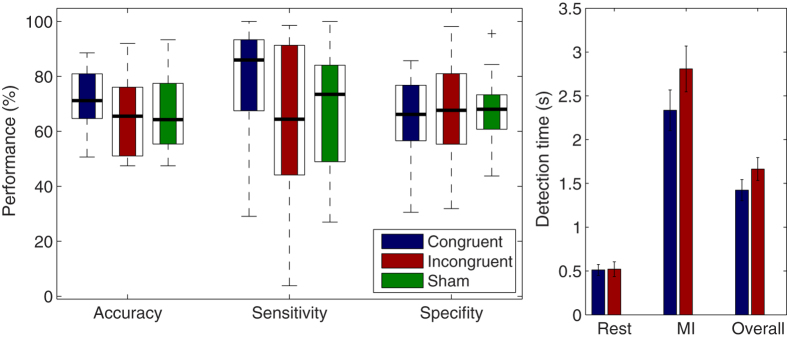
Performance analysis. Upper panel. Classification accuracies of each participant for each feedback phase. Asterisks indicate, whether the respecting classification accuracy was above the statistical chance (p = 0.05) level, or not. Lower left panel. Boxplots of median classification accuracies, sensitivity rates and specifity rates across subjects and for each feedback phase. Lower right panel. Mean detection times across subjects, separated for rest and MI periods.

Overall classification accuracies and detection times are depicted in Fig. 2. Classification accuracies were significantly above chance level (α = 0.05) in 48 of the 63 cases (76%). On average, the classification accuracies were 72% for the congruent feedback phase, 65% for the incongruent feedback phase and 67% for the sham feedback phase. A Friedman test revealed no significant effect for condition (Chi-quadrat = 4.09; df = 2, n = 21; p = 0.129). To get a better understanding of the classifier’s strengths and weaknesses, the classifier’s sensitivity and specificity rates were also calculated (see Fig. 2). The specificity rates were around 65% in all three conditions, whereas the sensitivity rate amounted to around 77% in the congruent, 69% in the sham and 64% in the incongruent feedback condition. A 3 × 2 repeated-measures ANOVA with the factors condition and performance (sensitivity, specifity) revealed a main effect of condition (F(1,20) = 6.02; p = 0.023) and an interaction (F(2,40) = 5.43; p = 0.030), but no main effect of performance (F(1,20) = 0.47; p = 0.500). The interaction was followed up by pairwise comparisons of the sensitivity rates, which revealed that sensitivity rates were higher in the congruent than incongruent condition (t(20) = 3.11; p = 0.005). No significant differences were found between congruent and sham (t(20) = 1.49; p = 0.150) or between incongruent and sham (t(20) = −0.713; p = 0.484).

### Detection times

Detection times were relatively low in the congruent (M = 0.51 s; SD = 0.28 s) and incongruent rest periods (M = 0.52 s; SD = 0.37 s) and higher in the congruent (M = 2.35 s; SD = 1.06 s) and incongruent MI periods (M = 2.80 s; SD = 1.19 s). A 2 × 2 repeated measures ANOVA revealed a main effect of period (F(1,20) = 4.52; p < 0.046) indicating that the detection times for the rest periods were lower than for the MI periods. Likewise, a main effect of condition (F(1,20) = 73.21; p < 0.001) was found indicating that the detection times were lower in the congruent than incongruent condition. In addition, a significant interaction between period and condition was found (F(1,20) = 4.38; p = 0.049). The interaction was followed up with post hoc t-tests. Significant effects were found for congruent-rest vs. congruent-move (t(20) = −7.59; p < 0.001), incongruent-rest vs. incongruent-move (t(20) = −7.97; p < 0.001), congruent-move vs. incongruent-move (t(20) = −2.21; p < 0.039), but not for congruent-rest vs. incongruent-rest (t(20) = −0.132; p < 0.896).

### Sense of Ownership

**Figure 3:**
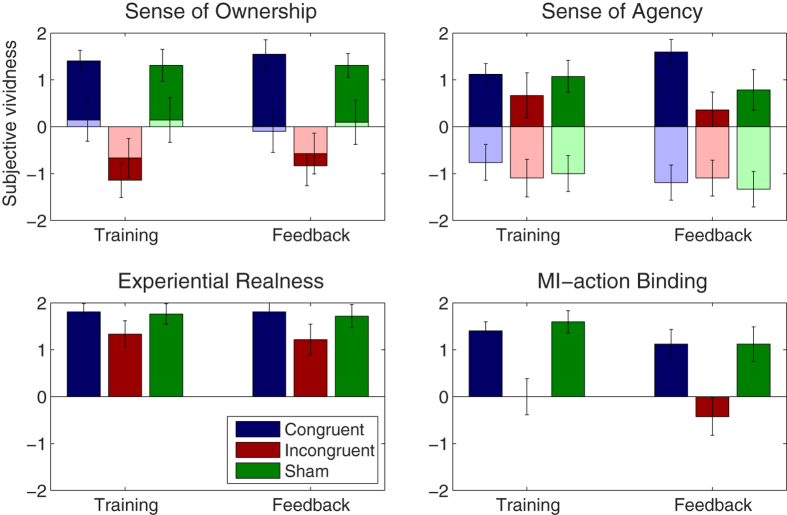
Subjective analysis. Mean (+SEM) questionnaire ratings for each experimental condition. For the sense of ownership and agency assessment, control questions were available. The results for these questions are indicated by light colors.

Questionnaire data are shown in Fig. 3. The a priori defined SoO induction criterion (+1) was met in the congruent train phase (M = 1.40; SD = 1.03), congruent feedback phase (M = 1.54; SD = 1.40), sham train phase (M = 1.30; SD = 1.56) and sham feedback phase (M = 1.30; SD = 1.17). Aversive illusion ratings were given for the incongruent train phase (M = −1.14; SD = 1.68) and incongruent feedback phase (M = 0.83; SD = 1.94). The t-tests between the SoO values and SoO control values were significant for the congruent (t(20) = 2.48; p = 0.017) and sham training condition (t(20) = 2.48; p = 0.017), but not for the incongruent training condition (t(20) = 0.85; p = 0.395). This confirms the illusion-specificity of the experimental manipulation. A 3 × 2 repeated measures ANOVA revealed a significant main effect for condition (F(2,40) = 39.49; p < 0.001), but no main effect for phase (F(1,20) = 0.57; p = 0.456) nor an interaction effect (F(2,40) = 0.43; p = 0.653). A planned t-test revealed a significant effect for congruent vs. incongruent conditions (t(20) = 7.14; p < 0.001), in that the SoO was reported to be higher in the two congruent conditions as compared to the two incongruent conditions. No significant effect was found for congruent feedback vs. sham feedback (t(20) = 1.05; p = 0.303).

### Sense of Agency

The a priori defined SoA induction criterion was reached in the congruent training phase (M = 1.11; SD = 1.03), congruent feedback phase (M = 1.59; SD = 1.22) and sham training phase (M = 1.07; SD = 1.58), but not in the incongruent train phase (M = 0.66; SD = 2.21), incongruent feedback phase (M = −0.35; SD = 1.76) or sham feedback phase (M = 0.78; SD = 1.98). The t-tests between the SoA values and SoA control values were significant for the congruent feedback (t(20) = 6.04; p < 0.001), incongruent feedback (t(20) = −2.67; p = 0.010) and sham feedback condition (t(20) = −3.67; p < 0.001). This indicates that under all forms of feedback, the participants clearly mentally isolated their SoA experience from other experiential forms of agency. A 3 × 2 repeated measures ANOVA revealed a significant main effect for condition (F(2,40) = 5.60; p = 0.007), but no main effect of phase (F(1,20) = 0.01; p = 0.911) nor an interaction effect (F(2,40) = 2.32; p = 0.111). A planned t-test revealed a significant difference between congruent and incongruent conditions (t(20) = 3.22; p < 0.004), reflecting that the SoA was higher in the two congruent as compared to the two incongruent conditions. Only a trend effect was found for congruent feedback vs. sham feedback (t(20) = 1.88; p = 0.075).

### Sham feedback detection

After the experiment, we disclosed to the participants that one of the two feedback conditions in which the hand was in anatomical alignment was a sham feedback condition. Fourteen of the 21 participants correctly identified the sham condition (67%; one-tailed binomial test: p = 0.094).

### Experiential Realness

ER was reported to be high (between 1.21 and 1.80) in all conditions. A 3 × 2 repeated-measures ANOVA with the factors condition and phase revealed a significant main effect of condition (F(2,40) = 5.71; p = 0.007), but no main effect of phase (F(1,20) = 0.13; p = 0.716) and no interaction (F(2,40) = 0.14; p = 0.866). A planned t-test revealed a significant difference between congruent and incongruent conditions (t(20) = 2.74; p < 0.013), in that the ER was reported to be higher in the two congruent conditions (M = 1.80; SD = 0.74) as compared to the two incongruent conditions (M = 1.27; SD = 1.31). No significant difference was found between congruent and sham feedback (t(20) = 0.56; p = 0.576).

### MI-action binding

MIAB was reported to be affirmative in the two congruent conditions (M = 1.26; SD = 0.95) and in the two sham conditions (M = 1.34; SD = 1.20) and to be around zero in the two incongruent conditions (M = −0.21; SD = 1.65). A 3 × 2 repeated-measures ANOVA revealed a significant main effect of condition (F(2,40) = 33.60; p = 0.001), but no main effect of phase (F(1,20) = 2.03; p = 0.169) and no interaction effect (F(2,40) = 0.27; p = 0.759). A planned t-test revealed a significant difference between congruent and incongruent conditions (t(20) = 5.54; p < 0.001), in that the MIAB was higher in the two congruent conditions as compared to the two incongruent conditions. No significant difference was found for congruent feedback vs. sham feedback (t(20) = 0.00; p = 0.999).

### Electrophysiological results

**Figure 4:**
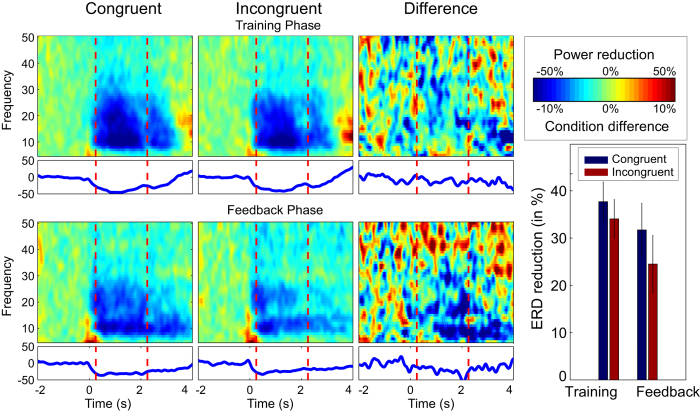
Time frequency analysis for congruent and incongruent conditions. Top panels. Time frequency plots showing percentage change in power from baseline for the training phase of the congruent condition (first column), incongruent condition (second column) and the difference (third column). Bottom panels. Time frequency plots for feedback phase showing congruent and incongruent conditions and the difference, as for the training phase. Vertical dashed red lines indicate time window of interest. Line plots below the time frequency plots indicate power averaged across frequencies from 10 to 25 Hz. Bottom right. Summary data averaged across latencies from 0.25 ms to 2.25 seconds and frequencies from 10 to 25 Hz.

ERD time frequency plots and ERD values across subjects are shown in Fig. 4. As can be seen from Fig. 4a, a clear reduction of ERD power from 8 to 30 Hz is evident at onset of the MI period. Thus, the expected ERD pattern was clearly evident in both congruent and incongruent conditions. A condition x phase repeated-measures 2 by 2 ANOVA revealed a main effect of condition (F(1,20) = 12.09; 0.002), with stronger ERD in the congruent than incongruent conditions. A main effect of phase (F(1,20) = 4.30; p = 0.051) was also found, with stronger ERD during the training phase as compared to the feedback phase. No significant interaction effect was found (F(1,20) = 2.78; p = 0.111).

### Phasic EDA

**Figure 5:**
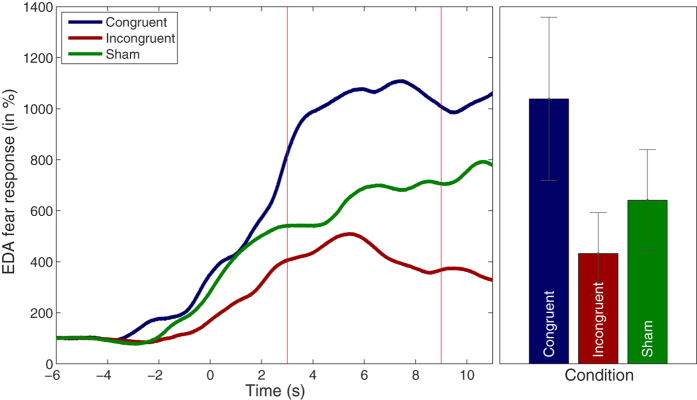
Phasic EDA responses. Left panel. Phasic EDA responses during syringe application. Although a phasic EDA response was observable in all conditions, the strongest increase (fear response) was clearly observed in the congruent feedback condition. Right panel. Mean phasic EDA response between 3 and 9 seconds.

Phasic EDA responses are shown in Fig. 5. As can be seen, a phasic increase of EDA shortly after the injection of the syringe was present in all three conditions. In the congruent condition the phasic EDA increase was 1038% (SD = 1318), in the incongruent condition 432% (SD = 664) and in the sham condition 640% (SD = 817). A one-way repeated-measures ANOVA revealed a main effect of condition (F(2,32) = 4.95; p = 0.013). Planned pairwise comparisons were conducted for each condition pair. A significantly stronger phasic EDA response was found in the congruent condition as compared to the incongruent condition, (t(16) = 2.67, p = 0.016). A trend effect was found for a stronger phasic EDA response in the sham condition than in the incongruent condition (t(16) = 1.87; p = 0.079), but no significant difference emerged between the congruent and sham condition (t(16) = −1.52; p = 0.148).

### Relationships between the measures

Significant correlations passing the Bonferroni adjustment (alpha = 0.0024) were found between SoO and ER (r = 0.793; p < 0.001), SoO and MIAB (r = 0.878; p < 0.001), SoA and ER (r = 0.740; p < 0.001), SoA and MIAB (r = 0.776; p < 0.001), and between ER and MIAB (r = 0.878; p < 0.001). Descriptively, high correlations were also found between SoO and SoA (r = 0.577; p = 0.006), detection time and SoA (r = −0.503; p = 0.020), and classification accuracy and detection time (r = −0.583; p = 0.005), but here the p-values did not survive Bonferroni correction. No significant correlations were found for any of the remaining measures.

## Discussion

We developed a new NF-MIT that employs an embodiable neurofeedback signal. Inspired by the RHI, we used an anthropomorphic robotic hand to visually guide the participant’s MI act and to deliver neurofeedback. Using two experimental manipulations, we investigated how the participants’ neurofeedback performance and subjective experiences were influenced, firstly, by embodiment of the robotic hand and, secondly, by the validity of the neurofeedback signal. In the following, the general feasibility of the study will first be briefly summarized and then the results of the two experimental manipulations discussed in detail.

The general feasibility of the present approach is shown in three ways. First, a SoO for the robotic hand could clearly be induced in 71% of our subjects. This shows that a RHI does not necessarily require synchronous stroking or movement of the artificial and participant’s real hand, but can also be induced by imagined limb movements in approximate synchrony to observed robotic-hand movements. This effect has so far only been systematically documented for a virtually-presented hand^21^,^46^, but it bears similarity to the movement illusions described for MVF^5^. Second, the expected ERD pattern of a right-handed MI task was evident in most individuals and served as the physiological basis for the classification algorithm. Third, in line with previous studies^18^, most individuals (76%) achieved a modest but higher than the statistical chance level online classification accuracy.

We systematically compared the effects of neurofeedback when provided by the movements of an anatomically congruently positioned robotic hand with those using an incongruently-positioned robotic hand. Clear effects of the embodiment manipulation were found in subjective, electrodermal, behavioral and electrophysiological measures, as described below.

Higher affirmative SoO ratings were given for the congruent than incongruent conditions. This confirms that anatomical alignment between the artificial and participant’s real hand is crucial to induce the illusion^32^,^33^,^34^,^53^. A novel result here is that this anatomical requirement also holds when the RHI is induced by limb movement imagination rather than by visuotactile stimulation^53^ or by motor execution^32^,^33^,^34^. Why an anatomical alignment is necessary to induce the illusion is still unresolved, but several theoretical accounts may apply (for a review, see ref. 54). The Bayesian causal inference model assumes that the RHI comes about by Bayesian sensory inference^36^,^55^. In its search for the most likely cause of its sensory input, the brain compares the evidence for whether its unimodal limb sensations have a common cause or independent causes, based on the similarity of sensations and the prior probability of a common cause. If the evidence for a common cause prevails, the disparate limb sensations are fused together and the RHI occurs^36^.

With regard to SoA, the participants rated their authorship experience over the robotic hand’s movements higher when it was anatomically aligned. Notably, this effect also occurred during the training phase where the robotic hand movements were identical between the congruent and incongruent conditions. The robotic hand position alone thus affected the SoA. A promoting effect of SoO on SoA has been previously reported^32^,^33^, but not in the context of movement imagination. Apparently, a neurofeedback signal facilitating embodiment not only produces a SoO experience, but also enhances the participant’s subjective experience of control over the neurofeedback signal.

With regard to MIAB, we are not aware of any previous RHI studies that have operationalized this construct before. With this measure, we evaluated how strongly the participants perceptually fused their self-induced MI percept with the robotic hand motion percept, in time and space. In our view, a complete fusion of percepts – i.e. an idealized scenario, where the mental act becomes phenomenally indistinguishable from the action achieved – would be indicative of a perfectly embodied neurofeedback signal (or brain-machine interface). None of our participants experienced such a complete perceptual fusion, but more MIAB was clearly reported for the congruent than incongruent conditions. This finding is compatible with the above-mentioned Bayesian framework, according to which perceptual fusion is more likely to occur the better the limb percepts match in time and space.

Our ER measure assessed how vividly and real the participants experienced their own MI act. Here we reasoned that on the subjective level, ER is the phenomenal target property that distinguishes motor execution from MI and that in a perfectly embodied NF-MIT (or brain machine interface), the MI act would be experienced as real, as if the mentally simulated movement was actually being executed. For this measure, participants also gave higher affirmative ratings for the congruent than incongruent conditions. This confirms our hypothesis that a participant’s MI experience can be enriched if it is accompanied by congruent robotic hand motion feedback – or by MVF. Assuming that the level of realness we attribute to a mental representation at least partly depends on its perceptual detail^56^, our explanation would be that the current finding may be attributed to the MIAB effect. Whereas in the congruent condition, the MI percept could be fused with the “high-resolution” robotic hand motion percept, and thus was enriched in perceptual detail, in the incongruent condition the MI-percept and robotic hand motion percept remained separate.

Our EDA results are also in support of a successful embodiment of the robotic hand, since the strongest phasic EDA response occurred in the congruent feedback condition. Human EDA is known to be under exclusive control of the sympathetic nervous system^57^. The sympathetic arousal was strongest in the congruent feedback condition, possibly because here the participants had incorporated the robotic hand into their own body schema and thus fearfully expected to experience a painful needle-prick. The interpretation of robotic hand embodiment in the congruent hand condition, as indicated by the EDA results, is also supported by the trend effect for a larger EDA response in the sham compared to the incongruent condition, since the hand was placed in a congruent condition during sham as well.

The neurofeedback performance was rather specifically modulated by the embodiment manipulation. The overall detection times were shorter in the congruent than incongruent condition, but the overall classification accuracies were not significantly different. In other words, the participants switched more quickly – but not with higher reliability – into the respective classifier state. Interestingly, a closer look into the period-specific performances revealed a more distinct embodiment effect. Whereas the specificity rates were similar between conditions, the sensitivity rates turned out to be higher in the congruent than incongruent condition. Likewise, whereas the resting period detection times were almost identical, the MI period detection times were shorter in the congruent than incongruent condition. These findings indicate that a congruent robotic hand motion feedback does not help to stay in a relaxation state, but it facilitates the MI act. This interpretation is in accord with our MIAB and ER findings, because during rest periods the robotic hand remained completely still and hence at this stage the two conditions did not systematically differ. Future studies should investigate how the embodiment effect behaves if during the rest period false positive feedback, i.e., robotic hand movement, is introduced.

Throughout the training and feedback phase, stronger ERDs were found during the MI periods in the congruent than incongruent conditions. Keeping in mind that the robotic hand movements occurred during the training phase, this finding illustrates how the ERD can be modulated by robotic hand motion feedback. Typically, an ERD occurs during MI, whereas during relaxation the sensorimotor rhythms idle^41^. The present finding fits well to the ER result of a more vivid MI experience in the congruent conditions and to the better MI-period detectability under congruent than incongruent feedback. It also confirms that the condition differences in neurofeedback performance were driven by the condition differences in the ERD and not by some classifier artifact.

Our second experimental manipulation pertained to the controllability of the neurofeedback signal. By contrasting real neurofeedback with sham feedback, we investigated whether the neurofeedback performances and subjective experiences were influenced by the validity of the neurofeedback signal. Relative to the embodiment manipulation, our findings for the controllability manipulation were more divergent. On the one hand, we found some indication that the participants experienced a higher SoA under actual than under sham feedback. When explicitly asked, 14 out of 21 participants (67%) correctly identified the sham feedback block. Likewise, stronger EDA increases were found under actual than under sham feedback. However, this ERD effect could also result from habituation, as the sham feedback always came after the congruent feedback. On the other hand, no other differences between the two forms of feedback were found in any of the remaining measures. Our overall interpretation is that, with the current experimental setting, it made little difference whether participants received real or sham feedback. It is possible that our sham feedback was too difficult to detect as such, as it was almost identical to the actual neurofeedback of the preceding congruent block. In fact, the only difference was that the real feedback depended on the current trial performance, whereas the sham feedback was based on the replayed performance of a past trial. Assuming that the participants faithfully followed the task instructions, the only chance to recognize that sham feedback was given was to detect a mismatch between the trial-by-trial-varying MI phenomenology and the trial-by-trial-varying robotic hand movement behavior.

The correlational analysis revealed consistently high correlations between all subjective measures. This suggests that our subjective measures did not relate to disparate, but rather to overlapping subsets of phenomenal experience and that they interacted with each other. In the following, we discuss some of the commonalities and interrelations between the different measures applied.

As regards the relationship between SoO and SoA, it has already been noted that the SoA is experienced more strongly if its phenomenal content is interpreted as part of the self. Given that all our actions originate in our body, although their impact often ranges beyond our body boundaries, it seems reasonable that the brain attributes higher certainty levels of authorship to our immediate body actions than to their less foreseeable effects on the world. Voluntary action however also seems to have an influence on the SoO^32^,^34^,^58^. An intuitive explanation, which fits well with the Bayesian brain idea^55^,^59^, would be that if the body is moved, the brain can test its predictions about what is part of the body and what is not.

An intrinsic relationship also appears to exist between MIAB and SoO, in that under certain circumstances MIAB may be important to enable SoO. According to the Bayesian account, the RHI occurs whenever the brain infers that its disparate limb sensations must have a common cause and consequently fuses them together. In our experiment, a perceptual fusion of the MI percept and robotic hand motion percept therefore seems crucial to induce the RHI.

With regard to the relationship between SoO and ER, we are not aware of any previous empirical studies dealing with this question, but an interesting theoretical account exists^60^,^61^. According to Metzinger’s self-model theory of subjectivity, both phenomenal experiences share a common requirement to be experienced, namely that their underlying mental representations have to be “phenomenally transparent”. A conscious mental representation is said to be transparent if its “vehicle properties” become introspectively inaccessible. As an example, consider the difference in phenomenal experience between MI and motor execution. Whereas in MI the subject of experience, in philosophical terminology, still remains conscious about the representational character of its mental representation, in motor execution it “forgets” that it is only dealing with a mental representation (i.e. the vehicle properties have become inaccessible). It therefore necessarily takes its representational content as something “irrevocably real”^60^ – namely a truly executed movement. The conceptual difference between ER and SoO is that whereas any transparent representational content (e.g. any sensory perception) can be experienced as real, a SoO may only arise for transparent self-representational content: “*The phenomenal property of selfhood is instantiated whenever a system has a conscious self-model that it cannot introspectively recognize as an internal model*”^60^. In our view, the SoO may thus be regarded as a special form of ER, namely as the irrevocable feeling of being the inhabitant of a body and of being in infinite closeness to this body.

In summary, this study encourages the development of embodied feedback signals for neurofeedback applications. Using a feedback signal that closely resembles the mental act performed may help to embody the feedback signal into the own body scheme and improve neurofeedback task-performance. Future studies should systematically test the role of embodied feedback signals on neurofeedback-guided motor rehabilitation.

## Additional Information

**How to cite this article**: Braun, N. *et al.* Embodied neurofeedback with an anthropomorphic robotic hand. *Sci. Rep.* **6**, 37696; doi: 10.1038/srep37696 (2016).

**Publisher’s note**: Springer Nature remains neutral with regard to jurisdictional claims in published maps and institutional affiliations.

## Figures and Tables

**Table 1:** Questionnaire for assessment of subjective experience.

Category	Statement
Sense of Ownership	I felt as if the robotic hand was my own hand.
	I felt as if the robotic hand was part of my body.
Sense of Ownership (control question)	I felt as if I no longer had a right hand, as if I my right hand had disappeared.
Sense of Agency	I felt as if I was controlling the movements of the robotic hand.
	Whenever I imagined a movement, the robotic hand started moving.
Sense of Agency (control question)	I felt as if the robotic hand were controlling my will.
Experiential Realness	My imagined movements felt as vivid and real as if they had actually happened.
	My imagined movements appeared as clear and detailed to my mind’s eye, as if they actually happened.
MI-action binding	I felt as if my imagined movements were happening at the position where the robotic hand was actually located.
	I experienced my imagined movements and the movements of the robotic hand to be inseparably linked with each other.
